# Supplementary material for: A Robust Discriminant Framework Based on Functional Biomarkers of EEG and Its Potential for Diagnosis of Alzheimer’s Disease
Source: Healthcare (Basel). 2020 Nov 11;8(4):476. doi: 10.3390/healthcare8040476 (PMC7712949; doi:10.3390/healthcare8040476)
Supplement: Supplementary file 1 [file healthcare-08-00476-s001.pdf]

Table S1. Discriminant results by different classifiers using 5-fold CV based on six candidate wavelet filters (%)

	modwt_filter	Rate (Mean ± SD)							
		LDA	Logreg	KNN	SVM	RF	Nbayes	Adaboost	NNet
Accuracy	Haar	91.75±4.01	92.11±2.05	72.76±13.82	84.15±10.76	81.81±8.97	80.18±8.34	83.16±8.84	91.68±3.21
	D4	90.44±3.20	91.80±3.51	77.21±8.12	88.12±4.95	81.37±8.23	81.84±7.38	81.61±7.97	89.33±4.48
	D6	92.21±4.36	90.45±3.45	75.93±8.42	86.52±5.76	78.72±9.23	79.09±8.34	78.14±7.48	87.38±5.32
	D8	91.61±3.94	91.59±2.00	71.36±7.58	84.53±6.23	78.51±8.71	78.22±7.37	80.44±6.40	91.47±1.98
	LA8	93.18±3.65	92.44±3.61	76.76±5.85	83.56±4.81	79.28±7.18	78.97±6.22	79.73±8.03	91.47±4.93
	C6	91.53±2.37	90.45±2.95	80.09±8.46	85.86±5.35	79.43±8.42	81.10±3.80	79.68±9.73	84.97±7.46
AUC	Haar	97.58±2.48	97.51±1.63	78.81±13.58	90.36±7.18	90.29±8.37	86.49±8.14	91.00±5.78	97.16±2.31
	D4	96.81±1.71	97.15±1.63	86.38±6.05	95.42±3.34	87.72±8.99	90.08±4.82	89.17±7.54	91.87±3.48
	D6	97.29±1.45	96.15±0.96	84.58±6.53	92.38±3.17	86.69±9.89	86.58±6.95	87.29±6.87	90.16±3.87
	D8	96.55±2.16	96.65±0.53	80.71±6.53	91.12±4.30	86.69±8.75	85.75±8.10	87.11±6.13	95.88±0.97
	LA8	97.92±1.66	97.18±1.77	86.69±4.61	91.28±3.99	88.05±7.12	89.12±3.81	87.40±6.85	96.60±1.84
	C6	97.15±1.06	96.74±1.03	88.88±6.21	93.34±4.05	86.70±9.64	89.80±3.22	86.90±8.85	88.34±5.88
Specificity	Haar	91.27±5.53	88.97±5.05	73.51±13.34	79.03±12.34	73.49±11.45	78.16±8.48	78.79±9.82	87.54±7.35
	D4	88.07±4.69	89.06±5.56	75.44±8.64	84.15±5.67	69.57±13.55	76.35±12.78	79.06±9.59	87.16±6.23
	D6	90.75±7.77	88.75±4.58	79.97±7.18	84.08±8.22	67.85±14.21	76.53±11.11	75.16±11.42	87.76±8.04
	D8	89.78±6.66	89.01±4.68	79.55±8.52	80.02±4.35	65.54±11.08	78.34±9.91	76.18±6.33	87.43±4.91
	LA8	91.45±6.98	88.04±9.71	75.81±2.76	81.18±7.78	66.30±8.43	85.68±9.14	75.41±11.00	85.02±12.13
	C6	88.23±6.47	88.68±5.00	79.23±7.36	82.37±6.96	69.38±12.68	79.16±5.59	75.52±7.44	82.99±9.60
Recall	Haar	93.20±5.30	94.57±2.28	72.59±14.44	89.35±10.08	89.86±9.21	78.54±11.39	87.98±10.36	95.77±2.79
	D4	92.20±3.52	93.46±2.80	77.67±12.15	90.96±5.39	89.52±6.21	84.96±6.23	83.66±13.88	90.09±3.63
	D6	93.67±2.71	90.35±4.50	74.41±13.28	88.60±5.53	86.57±8.45	77.56±11.08	82.7±7.42	85.67±9.57
	D8	92.72±2.32	92.35±2.61	67.59±14.13	87.58±7.85	87.55±7.49	75.61±10.34	84.28±7.10	93.09±2.75
	LA8	94.55±3.85	94.26±5.07	76.86±12.06	85.53±11.04	87.04±11.07	69.26±16.99	82.89±11.40	94.34±5.49

	C6	93.73±2.62	91.16±4.85	79.99±13.80	87.92±9.23	86.17±10.95	80.47±6.98	82.98±15.05	85.40±7.80
Precision	Haar	93.25±6.31	92.25±5.73	78.46±18.67	85.00±14.56	81.86±14.30	83.74±12.71	84.89±13.10	90.50±8.98
	D4	91.29±7.69	92.11±7.64	82.52±9.51	88.44±9.08	80.32±13.58	83.52±13.42	84.29±12.60	90.90±8.11
	D6	92.91±8.36	92.07±5.98	83.26±11.66	87.02±10.86	78.76±14.76	83.27±11.9	80.75±15.60	90.89±9.36
	D8	92.50±8.02	92.51±5.28	81.53±11.83	84.70±11.35	77.82±13.88	83.41±13.22	82.55±12.48	91.56±5.48
	LA8	94.02±7.95	93.05±8.32	81.85±10.45	85.85±11.43	78.78±12.77	88.88±6.89	82.00±13.85	91.76±9.50
	C6	92.02±7.93	91.89±7.36	84.66±8.36	87.19±9.42	79.28±14.45	84.50±9.80	81.46±13.13	87.2±13.10
F-measure	Haar	93.00±2.99	93.26±2.21	74.93±15.3	86.31±9.85	84.80±8.58	80.98±11.75	85.54±8.28	92.76±4.01
	D4	91.53±3.88	92.63±4.30	79.45±8.51	89.39±5.38	84.15±8.78	83.78±8.50	83.11±10.26	90.39±5.38
	D6	93.13±4.78	91.14±4.63	77.70±9.16	87.55±7.27	81.80±10.00	80.12±10.8	80.94±8.96	87.82±7.58
	D8	92.50±4.89	92.38±3.38	72.91±9.52	85.84±8.48	81.83±9.32	79.06±10.87	82.85±7.34	92.26±3.62
	LA8	94.06±4.04	93.36±4.31	78.79±9.21	84.92±7.74	82.01±9.49	77.28±13.11	81.69±10.21	92.68±5.18
	C6	92.62±3.28	91.31±4.20	81.67±9.18	87.08±6.55	81.92±10.76	82.36±7.97	81.50±11.61	85.88±9.20
